# Primary Tuberculosis of Tonsils: A Case Report

**DOI:** 10.1155/2012/120382

**Published:** 2012-03-11

**Authors:** Pooja Prasad, Minakshi Bhardwaj

**Affiliations:** Department of Pathology, PGIMER and Dr. Ram Manohar Lohia Hospital, 110001 New Delhi, India

## Abstract

Tuberculosis is one of the major causes of ill health and death worldwide. Isolated tuberculosis of tonsil in the absence of active pulmonary tuberculosis is a very rare clinical entity. A 10-year-male child presented with recurrent episodes of upper respiratory tract infections, with 2-3 occurrences per month for the past 6 years. On general physical examination, bilateral tonsils showed grade III enlargement and congestion. Posterior pharyngeal wall was clear. Examination of the chest was within normal limits. Histopathological examination of bilateral tonsils revealed caseating and noncaseating epithelioid cell granulomas with Langhans giant cells. Ziehl-Neelsen stain for acid fast bacillus was positive. Features were consistent with a diagnosis of tuberculosis of tonsils. Tuberculosis of the oral cavity is uncommon and lesions may be either primary or secondary. Early detection and intervention is essential for cure. Isolated and primary tuberculosis of the tonsils in the absence of pulmonary tuberculosis is a rare entity, which prompted us to report this case.

## 1. Introduction

Tuberculosis is one of the major causes of ill health and death worldwide. Primary tuberculosis of the oral cavity and oropharynx is quite uncommon. Isolated tuberculosis of tonsil in the absence of active pulmonary tuberculosis is a very rare clinical entity [1, 2]. We report a case of primary tonsillar tuberculosis, in an otherwise healthy child, mimicking chronic nonspecific tonsillitis.

## 2. Case Report

A 10-year-male child presented with recurrent episodes of upper respiratory tract infections, with 2-3 occurrences per month for the past 6 years. The patient had cough and cold associated with fever and difficulty in swallowing. There was a history of snoring, mouth breathing, and sleeping in the prone position. Past and family history was not significant. The child had been on antibiotic treatment for the previous episodes, but did not respond to them. Family history was not significant.

On general physical examination, the child was of healthy build with bilateral level IIIb cervical lymphadenopathy. On oral examination, bilateral tonsils showed grade III enlargement and congestion. Posterior pharyngeal wall was clear. Examination of the chest was within normal limits.

Routine investigations revealed Hb-13 g%, TLC-5800/mm^3^, and ESR-6 mm. Liver and renal function tests were normal. Mantoux test was positive with 18 × 20 mm induration. X-ray of the chest was within normal limits. The patient was HIV seronegative. Fine-needle aspiration of the lymph nodes revealed features of reactive hyperplasia, with stain for acid fast bacillus being negative.

**Figure 1:**
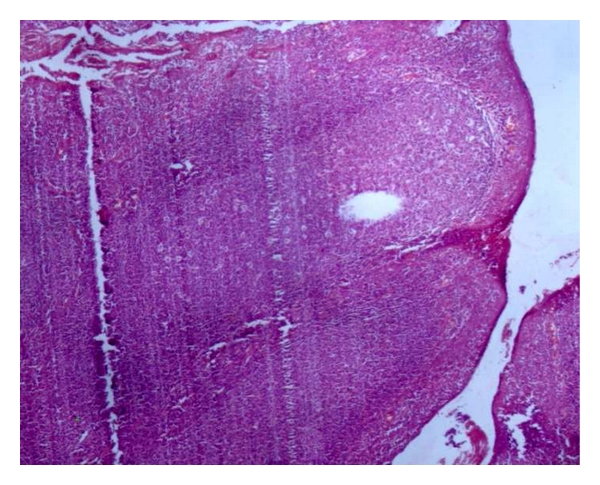
Tonsillar architecture (H&E 4x).

**Figure 2:**
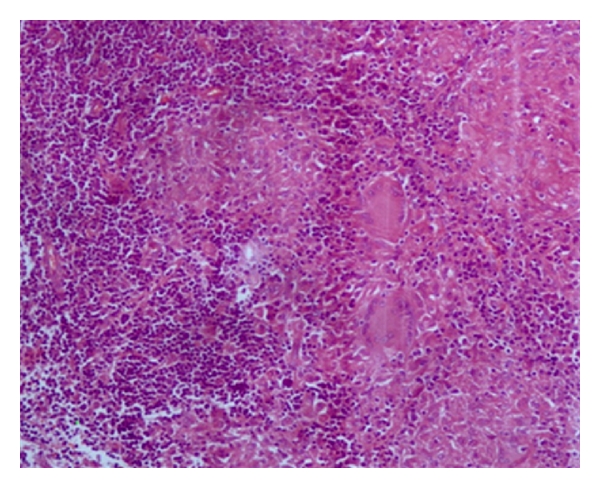
Noncaseating epitheliod cell granulomas (H&E 100x).

**Figure 3:**
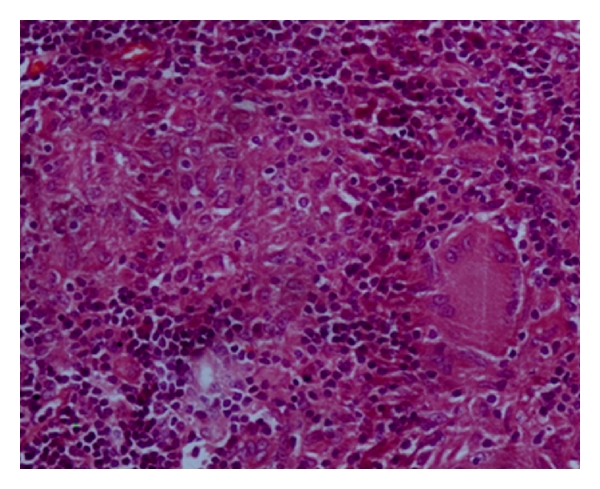
Noncaseating epitheliod cell granulomas (H&E 400x).

**Figure 4:**
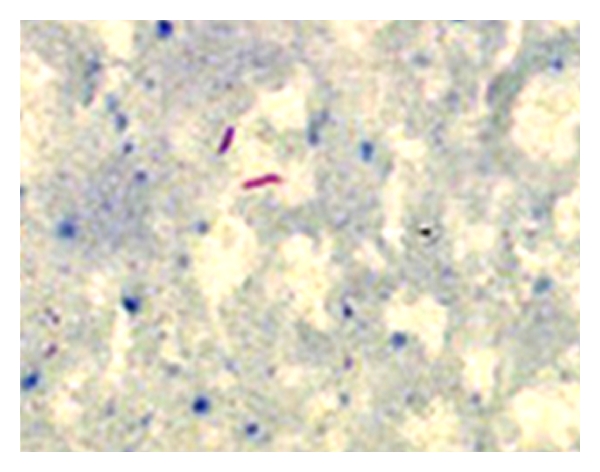
Acid fast bacilli (ZN oil immersion).

The child underwent tonsillectomy, for a clinical diagnosis of chronic tonsillitis. Histopathological examination of bilateral tonsils revealed caseating and noncaseating epithelioid cell granulomas with Langhans giant cells (Figures 1, 2, and 3). Ziehl-Neelsen stain for acid fast bacillus was positive (Figure 4). Features were consistent with a diagnosis of tuberculosis of tonsils.

The patient was treated with 2HRZE/4HR regimen of Isoniazid (300 mg), Rifampicin (450 mg), Ethambutol (800 mg), and Pyrazinamide (1500 mg) on alternate days thrice a week for two months, followed by Rifampicin (450 mg) and Isoniazid (300 mg) on alternate days, thrice a week for the next four months. The patient showed marked improvement in symptoms and became asymptomatic within two months. The child is presently under followup to complete the prescribed regimen.

## 3. Discussion

Extrapulmonary tuberculosis (TB) represents approximately 25% of overall tubercular morbidity [3]. Among extra pulmonary tuberculosis (EPTB), most common is lymph node tuberculosis while other forms are pleural tuberculosis, skeletal tuberculosis, CNS tuberculosis, abdominal tuberculosis, genitourinary tuberculosis, and miliary tuberculosis, tubercular pericarditis is also seen.

Tuberculosis of the oral cavity is uncommon and lesions may be either primary or secondary. Tongue and palate are the common sites whereas tonsillar tuberculosis is a rare localization with an incidence of less than 5% [4]. Tuberculosis of the tonsil can result from infection by contact with material containing tubercle bacilli. Tonsillar TB commonly presents with sore throat and cervical lymphadenopathy. 

Miller [5] in 1963 concluded that with the advent of pasteurized milk the incidence of tuberculosis came down still further. Tonsil is made up of lymphoid tissue and is situated at a site which is frequently drenched with infected sputum. Still tuberculous infection of tonsil is a rarity because of the antiseptic and cleansing action of saliva, inherent resistance of tonsil to tuberculous infection, presence of saprophytes in the oral cavity making colonization difficult and the thick protective stratified squamous epithelial covering over tonsil [6].

Although tuberculosis of tonsil is now an uncommon finding, tonsillar granulomata are commonly seen in patients with poor host reaction due to alcoholism, HIV infection, and so forth. Predisposing factors for primary oral tuberculosis include poor dental hygiene, dental extraction, periodontitis, and leucoplakia. It has been postulated that such infections are acquired by inhalation, with harbouring of disease in Waldeyer's ring. Differential diagnosis of oral and pharyngeal tuberculosis includes traumatic ulcers, aphthous ulcers, hematological disorders, actinomycosis, syphilis, midline granuloma, Wegner's disease, and malignancy [7]. Diagnosis of tonsillar tuberculosis is based on histopathological findings and the identification of tubercle bacilli. Treatment is in the form of antituberculosis therapy.

## 4. Conclusion

Tuberculosis of the tonsils might be suspected if the tonsils are enlarged unequally on the two sides and are associated with cervical lymphadenopathy. Patients seek medical advice due to sore throat and difficulty in deglutition. Tuberculosis is a severe and life-threatening disease. The number of new cases is increasing in both developed and developing countries. Early detection and intervention is essential for cure. Isolated and primary tuberculosis of the tonsils in the absence of pulmonary tuberculosis is a rare entity, which prompted us to report this case.

## References

[1] Weidman WN, Campbell HB (1939). Laryngeal tuberculosis. *American Review of Tuberculosis*.

[2] Kant S, Verma SK, Sanjay (2008). Isolated tonsil tuberculosis. *Lung India*.

[3] Farer LS, Lowell AM, Meader MP (1992). Extra pulmonary tuberculosis in USA. *American Journal of Epidemiology*.

[4] Wilkinson HF (1929). A study of ten thousand pairs of tonsils, with special reference to the presence of cartilage, bone, tuberculosis and bodies suggestive of actinomycosis. *Archives of Otolaryngology*.

[5] Miller FJ, Seal W, Taylor MD (1963). *Tuberculosis in Children*.

[6] Jana U, Mukherjee S (2003). Tuberculosis of tonsil—a rare site involvement. *Indian Journal of Otolaryngology and Head and Neck Surgery*.

[7] Gupta KB, Tandon S, Jaswal ST, Singh S (2001). Tuberculosis of the tonsil with unusual presentation. *Indian Journal of Tuberculosis*.

